# A method for labeling proteins with tags at the native genomic loci in budding yeast

**DOI:** 10.1371/journal.pone.0176184

**Published:** 2017-05-01

**Authors:** Qian Wang, Huijun Xue, Siqi Li, Ying Chen, Xuelei Tian, Xin Xu, Wei Xiao, Yu Vincent Fu

**Affiliations:** 1 College of Life Sciences, Capital Normal University, Beijing, China; 2 State Key Laboratory of Microbial Resources, Institute of Microbiology, Chinese Academy of Sciences, Beijing, China; 3 Savaid Medical School, University of Chinese Academy of Sciences, Beijing, China; 4 National Institute for Radiological Protection, China CDC, Beijing, China; 5 Department of Microbiology and Immunology, University of Saskatchewan, Saskatoon, SK Canada; Florida State University, UNITED STATES

## Abstract

Fluorescent proteins and epitope tags are often used as protein fusion tags to study target proteins. One prevailing technique in the budding yeast *Saccharomyces cerevisiae* is to fuse these tags to a target gene at the precise chromosomal location via homologous recombination. However, several limitations hamper the application of this technique, such as the selectable markers not being reusable, tagging of only the C-terminal being possible, and a “scar” sequence being left in the genome. Here, we describe a strategy to solve these problems by tagging target genes based on a pop-in/pop-out and counter-selection system. Three fluorescent protein tag (mCherry, sfGFP, and mKikGR) and two epitope tag (HA and 3×FLAG) constructs were developed and utilized to tag *HHT1*, *UBC13* or *RAD5* at the chromosomal locus as proof-of-concept.

## Introduction

The budding yeast *Saccharomyces cerevisiae* has been widely used in basic research due to the ease with which it can be genetically manipulated. Considerable progress has been made in studies on budding yeast via the use of epitope and fluorescent protein (FP) tags. Epitope tags, such as Flag, HA, and Myc, are useful for western blotting analysis, immunoprecipitation, affinity purification, and immunochemistry [1–3]. FP tags, such as green fluorescent protein (GFP) and its variants, are extensively used in molecular and cellular biology studies. In many experiments, an FP has been fused to a target protein as a reporter to track its cellular dynamics. For instance, the global protein subcellular localization was studied by plasmid-based epitope-tagging and immunofluorescence analyses [4], or by fusing a GFP tag at the C-terminal of the coding region of a gene of interest, followed by microscopic imaging [5].

Integration of an epitope or FP tag into the desired chromosomal locus is particularly easy to carry out in *Saccharomyces cerevisiae* due to the ease of introducing a tag sequence into the genome by homologous recombination (HR). Compared with the plasmid-based protein-tagging method, chromosomal gene-tagging has higher genetic stability and ensures that there is one copy of the fusion gene in a haploid yeast cell. Moreover, chromosomal gene-tagging can avoid numerous artifacts since the fusion gene is in its native chromosomal context and expressed under the control of its native promoter. In recent decades, techniques have been developed that enable directed insertion of desired genetic elements into a precise location in a budding yeast chromosome. The general process in these techniques involves integrating a given element along with a selectable marker gene into a chromosome via HR, obtaining transformants on corresponding selective medium, and screening for the desired recombinant product by PCR of genomic DNA. Two types of marker gene have been used for selection. One is an auxotrophic marker gene, including *TRP1*, *LEU2*, *ADE2*, *MET15* [6], *HIS3* [7], *LYS2* [8], and *URA3* [9]. The other is a dominant marker gene, such as *KanMX* [10], *natMX* [11], and *amdSYM* [12].

For auxotrophic markers, *URA3* is the most widely used recyclable marker, although *LYS2* and *MET15* can be counter-selected by alpha-aminoadipate and methyl-mercury, respectively [8, 9, 12, 13]. Alani et al. successfully developed a pop-in/pop-out system for recycling the *URA3* marker as follows. A 3.8 kb DNA fragment consisting of *URA3* flanked by direct repeats of 1.1 kb *hisG* from *Salmonella* (*hisG*-*URA3*-*hisG*) was integrated into the yeast chromosome. The Ura^−^ transformants resulting from popping out of the *URA3* gene along with one copy of *hisG* were counter-selected in the presence of 5-fluoroorotic acid (5-FOA) [9, 14, 15]. However, in this system, one unwanted copy of *hisG* still remains in the yeast genome after the *URA3* gene has been popped out, which produces “scar” sequences around the target gene.

Using the dominant markers, it is easy to add a tag at the C-terminal of a gene of interest at its native chromosomal location. However, almost none of these markers are counter-selectable. A *Cre/loxP* recombination system was thus developed to make dominant markers reusable by flanking the markers with *loxP* [16, 17]. However, similar to the problem in the *URA3* pop-in/pop-out system, the *Cre/loxP* recombination system leaves one copy of *loxP* in the chromosome. Subsequently, the *amdSYM* cassette was reported as the first recyclable dominant marker through counter-selection with the acetamide homologue fluoroacetamide[18]. Actually, this new dominant recyclable marker has not been widely used as *URA3* marker.

PCR-based epitope tagging using plasmids containing tag-URA-tag as templates in budding yeast have been introduced firstly in 1995[19]. YFGs were tagged with 3×HA and 3×MYC, and the tagged proteins were verfied by western blotting. Then four plasmids containing triple-epitope tags (FLAG, HSV, V5 and VSV-G) were constructed in the same strategy[20]. General primer sequences were provided in the two papers to allow tagging the YFGs with all these epitopes using a single pair of primers, while a short nucleotide fragment will be left in the genome.

In our previous study, we reported a method to integrate a designed promoter into a target locus on a chromosome without introducing any junk sequence [21]. Here, using the same strategy, we fused FP tags (mCherry, sfGFP, and mKikGR) to a yeast gene (*UBC13*, *RAD5* or *HHT1*) and monitored FP-fused protein expression. We tagged *UBC13* with epitope tags (HA and 3×FLAG) and analyzed the results using western blotting. This study provides a reliable protein-tagging method, which is characterized by the recycling of selectable markers, ease of tagging the N-terminal of nonessential genes, and a minimal disturbance of native gene expression level.

## Materials and methods

### Plasmid construction

To obtain the three fluorescent protein tag constructs, pCUC, psfGUG, and pKUK, pBS-URA3 was constructed by first inserting *URA3* into pBluescriptII SK between the *Hin*dIII and *Eco*RI sites. Then, 3′-truncated and 5′-truncated mCherry, sfGFP, and mKikGR were cloned into the *Xho*I-*Hin*dIII site and the *Eco*RI-*Bam*HI site in pBS-URA3, respectively. For the two peptide tag constructs, pHUH and pFUF, *URA3* flanked by HA and that flanked by 3×FLAG were integrated into the *Hin*dIII-*Eco*RI and *Xho*I-*Bam*HI sites of pBluescriptII SK, respectively.

### Yeast strains

In this study, all strains were isogenic derivatives of strains BY4741 (Invitrogen), HK578-10A (H. Klein), HK578-10D (H. Klein), and yRH182[2]. *HHT1*, *UBC13* and *RAD5*, shown as your favorite genes (YFGs), were targeted for tagging through HR. To increase the efficiency of integration into the yeast genome, ~300-bp homologous sequences from both up- and downstream of each open reading frame (ORF) were added to both ends of *FP-URA3-FP* by overlapping PCR. The *5’ YFG-FP-URA3-FP-YFG 3’* fragments were amplified and transformed into yeast strains via a LiAc/SS carrier DNA/PEG method, as described previously [22]. The colonies that could grow on plates with SD-Ura medium (synthetic dextrose medium without uracil) were selected and then patched onto 5-FOA plates [14] to obtain marker-free single colonies, as described previously [9]. Genomic PCR and sequencing were used to confirm the correct tagging of YFG of the resulting strains. All of the primers used for plasmid construction, protein tagging, and strain verification are listed in S1–S3 Tables.

### Fluorescence microscopy

The yeast strains were cultured overnight at 30°C in synthetic complete medium, and were subcultured in fresh medium for about 4 h until the phase of exponential growth was reached. One milliliter of the culture was then collected by centrifugation at 3,000 *g* for 1 min, and the pellet was resuspended in 20 μL of phosphate-buffered saline. A total of 1 μg/mL DAPI (D9542, Sigma-Aldrich) was used to stain the nucleus for 10 min at room temperature. Fluorescence was detected by laser scanning confocal microscope (Zeiss LSM780), using excitation at 405 nm for DAPI, 488 nm for sfGFP and green mKikGR, 561 nm for mCherry, and 594 nm for red mKikGR. The images were processed by ZEN software.

### Western blot analysis

Protein extracts were prepared using the YeastBuster^TM^ Protein Extraction Reagent (71186, Merck Millipore). The antibodies used in this study were HA-probe antibody (F-7) (sc-7392, Santa Cruz Biotechnology), monoclonal anti-Flag M2 antibody (F1804, Sigma-Aldrich), and anti-Pgk1 (a kind gift from Wei Li, Institute of Zoology, Chinese Academy of Sciences).

## Results

### Construction of FP- and epitope-tag-integrating cassettes for tagging at the native genomic locus

**Fig 1:**
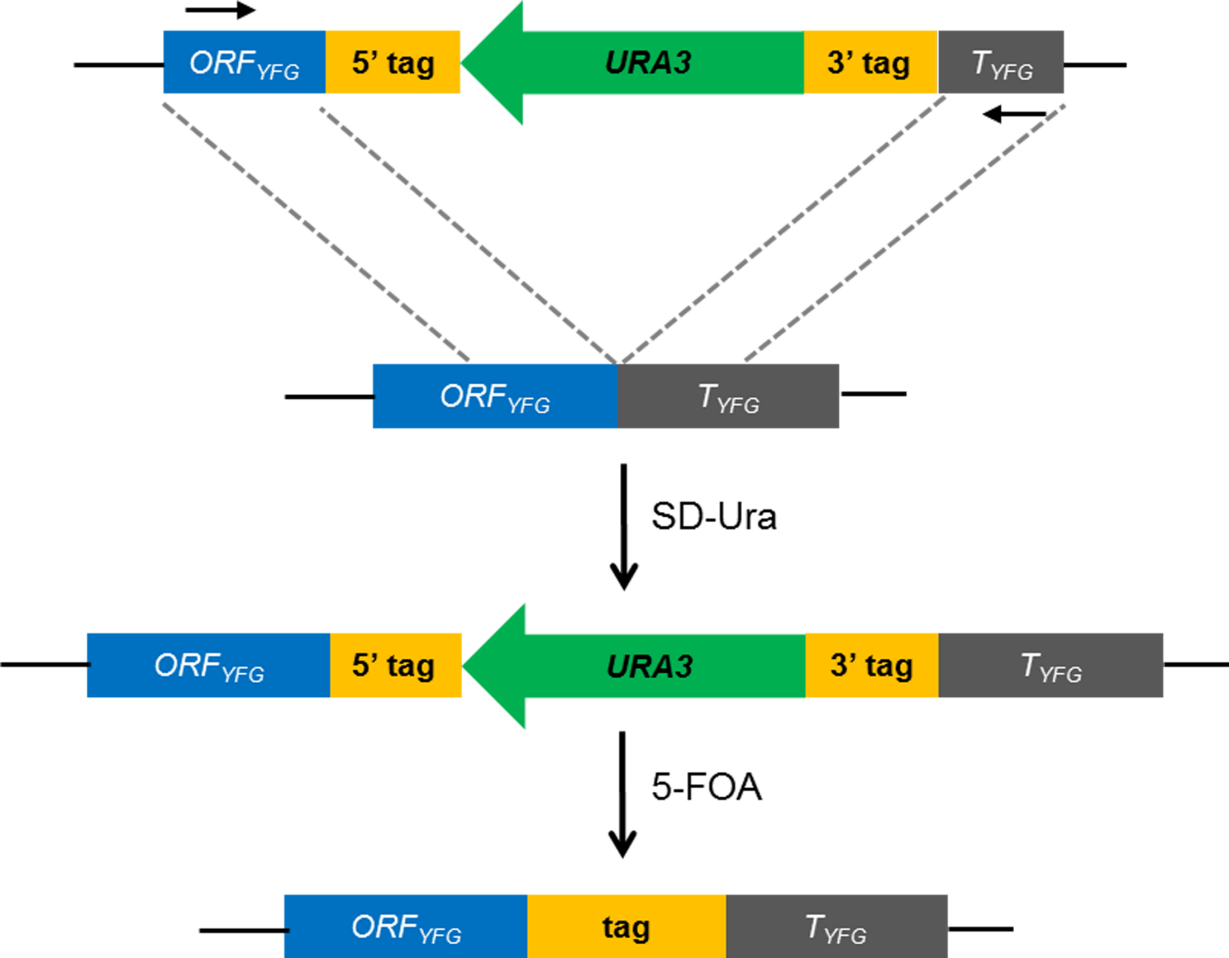
Schematic representation of the protein-tagging via the *URA3* gene pop-in/pop-out system. Here, we use tagging of YFG C-terminal as an example, and the N-terminal tagging of nonessential genes could be done following the same strategy. First, the *ORF*_*YFG*_*-5′tag-URA3-3′tag-T*_*YFG*_ fragment is integrated into yeast chromosomes through HR using the homologous sequences of the ORF and terminator of *YFG*. Positive selection on an SD-Ura plate yields pop-in clones in which the designed sequence has been successfully integrated into the chromosomal locus of the *YFG* gene. Second, the *URA3* gene would be eliminated via homologous recombination due to the duplicated sequences of 5′ and 3′ tags, leaving only one copy of the intact tag. The strains in which *URA3* has been popped out are counter-selected on 5-fluoroorotic acid plates.

Labeling a gene of interest with a tag at its native locus in the chromosome is a commonly used technique in studies on budding yeast. To solve the two main problems of an inability to recycle the selectable marker and the presence of “scar” sequences after tagging, we proposed that the *URA3* flanking sequences *hisG* in the traditional *hisG-URA3-hisG* system [9] could be replaced by the desired genetic element in tandem repeats. Thus, after site-specific integration and pop-out, only the designed element will remain at the predetermined locus, as illustrated in Fig 1.

Here, we designed a system to use FP sequences to replace *hisG* to form *FP-URA3-FP* cassettes. After HR between flanking homologous FP sequences, the *URA3* gene along with one copy of the repeat FP sequence was removed and formed an intact FP sequence at the target locus. To validate this concept, we chose three typical FPs: mCherry, sfGPF (superfolder GFP), and mKikGR, as tags. mCherry is a red monomer that is widely used due to it having the best photostability among the red FPs [23]. sfGFP is an engineered GFP with increased resistance to denaturation and improved folding kinetics [24]. Finally, mKikGR is a photoswitchable fluorescent protein that emits green fluorescence in its initial state, which is converted to red fluorescence after illumination with a 405-nm laser [25].

**Fig 2:**
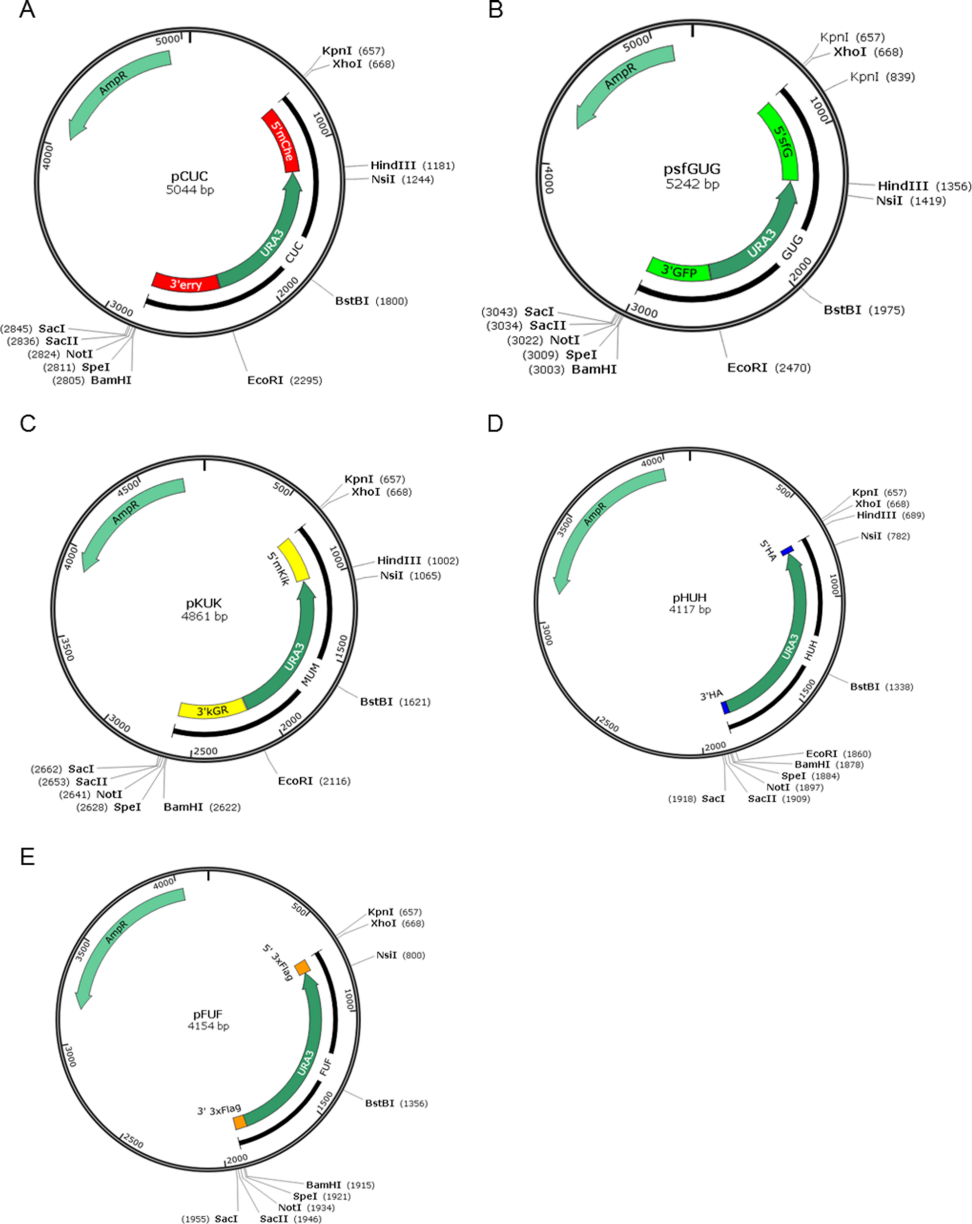
Plasmid constructs for protein-tagging. The original plasmid is pBluescriptII SK, and the maps display the restriction enzyme sites used for construction. (A) The pCUC map contains the 5’ *mChe-URA3-*3’ *erry* cassette. (B) The psfGUG map contains the 5’ *sfG-URA3-*3’ *GFP* cassette. (C) The pKUK map contains the 5’ *mKik-URA3-*3’ *kGR* cassette. For pCUC, psfGUG and pKUK, each pair of sectional tag sequences has a partially duplicated sequence for homologous recombination. (D) The pHUH map contains the 5’*HA-URA3-*3’*HA* cassette. (E) The pFUF map contains the 5’*3*×*FLAG-URA3-*3’*3*×*FLAG* cassette. For these two plasmids, the HA and FLAG tags are too short to be split, so the full-length sequences are used for homologous recombination.

To construct these three *FP-URA3-FP* cassettes, the *URA3* marker gene was first introduced into pBluescriptII SK using *Hin*dIII and *Eco*RI. Based on this new construct, a 5′ section containing C-terminally truncated FP and a 3′ section lacking N-terminal sequences of FP were cloned into *Xho*I-*Hin*dIII sites and *Eco*RI-*Bam*HI sites, respectively, flanking the *URA3* to generate three plasmids: pCUC, psfGUG, and pKUK (Fig 2). In each case, both FP coding sequences were truncated at either the 5′ or the 3′ end (Table 1), instead of using the full-length sequences, for three main reasons. First, compared with an epitope or affinity tag, FP coding sequences are much longer, while only about 50-bp homologous sequences are considered necessary for efficient popping out through HR. Second, differential truncation of the tandem repeats facilitates the selection of unique sequences on each copy to amplify the cassette for transformation by PCR. Third, since neither copy of the tandem repeats is intact, the initial integration product is negative for fluorescence, and only correct pop-out strains can display fluorescence. Accordingly, it is convenient to screen and confirm the desired final product.

**Table 1:** Truncation sequences of fluorescent proteins tags flanked by the *URA3* gene.

Fluorescent protein	Full length (bp)	5’ section (bp)	3’ section (bp)	Overlap section (bp)
**mCherry**	711	1–507	205–708	303
**sfGFP**	720	1–676	200–717	477
**mKikGR**	699	1–322	203–696	120
**HA**	30	1–30	1–30	30
**3×FLAG**	69	1–69	1–69	69

In addition to the FP tags, two plasmids containing URA3 flanking with full length HA and 3×FLAG tags were constructed, and named as pHUH and pFUF, respectively (Table 1, Fig 2).

### Verification of FP-tagging at the native genomic locus using microscopic imaging

**Fig 3:**
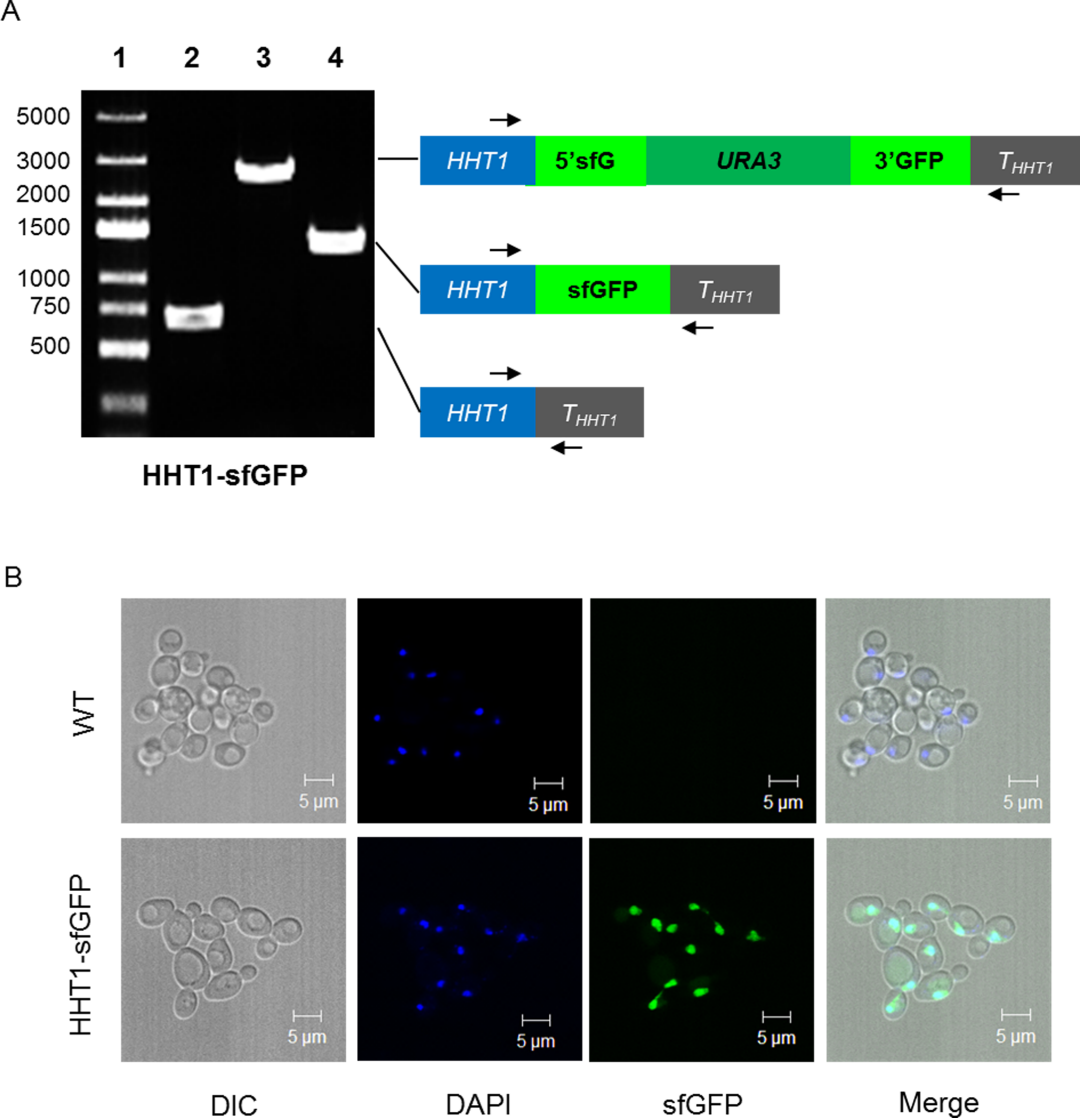
PCR analysis of tag-*HHT1* yeast strains and their subcellular localizations. (A) HHT1-sfGFP strain. The genomic DNA PCR data shows in the left panel. Lane 1: DNA ladder marker. Lane 2: the original strain yRH182. Lane 3 and 4: the pop-in and pop-out strain. (B) The subcellular localizations of HHT1-sfGFP. The yRH182 (upper) is the wild-type yeast strains without modification; the tagged strains *HHT1*-sfGFP are shown at the bottom. The images were obtained under Plan-Apochromat 63×/1.40 oil (Zeiss 5 Live).

**Fig 4:**
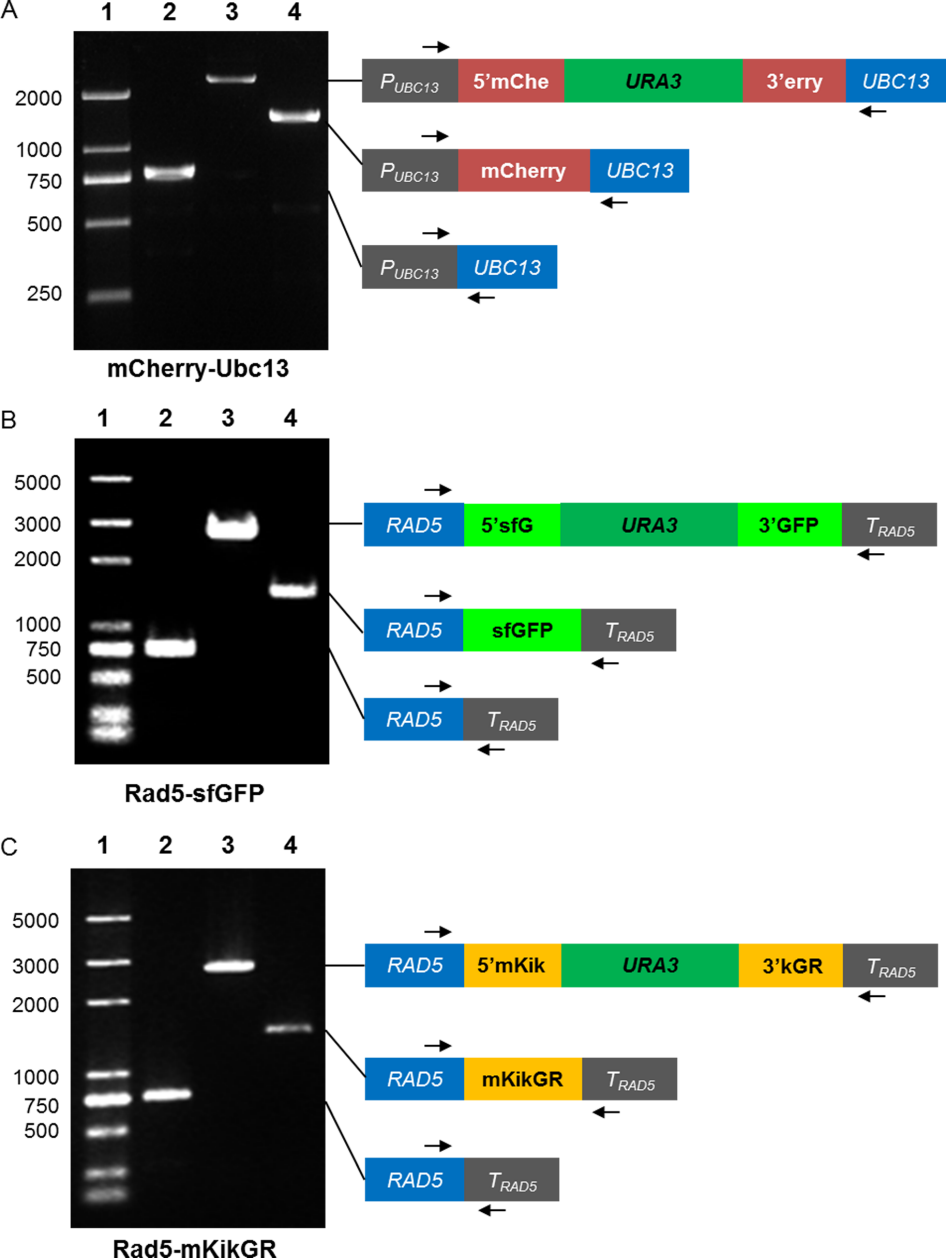
PCR analysis of tag-*UBC13*/*RAD5* yeast strains. (A) mCherry-Ubc13 strain. The left panel displays the genomic DNA PCR data. Lane 1: DNA ladder marker. Lane 2: the original strain HK578-10A. Lane 3: the pop-in strain CUC-Ubc13. Lane 4: the pop-out strain mCherry-Ubc13. The right panel represents the corresponding genomic organizations at the locus of *UBC13* in these three different strains. (B) Rad5-sfGFP strain. The original strain is HK578-10D. (C) Rad5-mKikGR strain. The original strain is HK578-10D. Arrows indicate the locations of primers. The detail information of primers could be found in the part of yeast strains check primers in S3 Table.

As the proof-of-concept, we aimed to tag chromosomal *HHT1*, *UBC13* and *RAD5* genes with three FPs. sfGFP was fused to the C-terminal of *HHT1*, which encodes one of two identical histone H3 proteins[26]. Based on the fact that the N-terminal fusion may affect the physical interaction of Rad5 with Rev1 [27, 28], the mKikGR tags were tethered to the C-terminal of Rad5, while mCherry was fused to the N-terminal of Ubc13. About 0.3~0.4 kb homologous sequences upstream and downstream of the locus of insertion were introduced into the *FP-URA3-FP* cassettes to generate *5′YFG-FP-URA3-FP-YFG3′* products by overlap PCR (S1 Fig). Table 2 lists the primers used to amplify each cassette sequence followed by gene-specific sequences, and their relative reading frame. These *5′YFG-FP-URA3-FP-YFG3′* products were used to transform yeast strains. After selection by SD-Ura, followed by counter-selection with 5-FOA, the resulting strains (Table 3) containing the desired FP fusion proteins were generated. Fig 3A shows the results of PCR analysis of the resulting *HHT1-sfGFP* strain and intermediates, while Fig 4 shows that of *RAD5-sfGFP* and *RAD5-mKikGR* strains.

**Table 2:** Proteins tagging primers used in this study^ɑ^.

Primers	Sequences (5’→3’)
**HA-Ubc13**	
pNUbc13-HUH F	TTAGCAAATAAGGTCAGGTTCATTGTAACATAGTTAGAA ATGTACCCATACGATGTTCC
pNHUH-Ubc13 R	TGAACATACCTTGATTATTCTCTTGGGTAATGATGCCAT AGCGTAATCTGGAACATCGT
**UBC13-HA**	
pCUbc13-HUH F	CGCGAATGGACGAAATTGTATGCAAAGAAGAAACCCGAG ATGTACCCATACGATGTTCC
pCHUH-Ubc13 R	TTATATATTCAGTTGAGAAAACTTATACAGAAATGATCA AGCGTAATCTGGAACATCGT
**3**x**FLAG-Ubc13**	
pUbc13-FUF F	TTAGCAAATAAGGTCAGGTTCATTGTAACATAGTTAGAA ATGGACTACAAAGACCATGA
pFUF-Ubc13 R	TGAACATACCTTGATTATTCTCTTGGGTAATGATGCCAT CTTGTCATCGTCATCCTTGT
**mCherry-Ubc13**	
pUbc13 F pUbc13-mCherry R pUbc13-mCherry F	AGCTTAATGATTAGATTCTG TCCTCGCCCTTGCTCACCATTTCTAACTATGTTACAATGA TCATTGTAACATAGTTAGAAATGGTGAGCAAGGGCGAGGA
pmCherry-Ubc13 R pmCherry-Ubc13 F	CTCTTGGGTAATGATGCCATCTTGTACAGCTCGTCCATGC GCATGGACGAGCTGTACAAGATGGCATCATTACCCAAGAG
pUbc13 R **Rad5-sfGFP** pRad5 F pRad5-sfGFP R	AAGTAACGTAAGTTGTCGTC GCCGTTAAAGACTATAGCAG AGCTCTTCGCCTTTACGCATTTCAAACAGCATCTGGATTTC
pRad5-sfGFP F psfGFP-Rad5 R psfGFP-Rad5 F	GAAATCCAGATGCTGTTTGAAATGCGTAAAGGCGAAGAGCT TATGAGTATGTGGTATGACTA TCATCATTTGTACAGTTCATCCATA TATGGATGAACTGTACAAATGATGATAGTCATACCACATACTCATA
pRad5 R	CCACCTACACTTTCTGTCAT
**Rad5-mKikGR**	
pRad5-mKikGR F pRad5-mKikGR R pmKikGR-Rad5 F	GAAATCCAGATGCTGTTTGAA ATGAGTGTGATTACATCAGA TCTGATGTAATCACACTCATTTCAAACAGCATCTGGATTTC GGGCGCCAAGTATGAATTTGAAGCCTAGTCATACCACATACTCATA
pmKikGR-Rad5 R	TATGAGTATGTGGTATGACTA GGCTTCAAATTCATACTTGGCGCCC
**HHT1-sfGFP**	
pHHT1-sfGFP F	GATATCAAGTTGGCTAGAAGATTAAGAGGTGAAAGATCA ATGCGTAAAGGCGAAGAGCT
pHHT1-sfGFP R	GTTCGTTTTTTACTAAAACTGATGACAATCAACAAACTA TCATCATTTGTACAGTTCATCCATA

^***ɑ***^ Primers used to amplify each cassette are listed here. Each primer consists of two parts. One part is the sequence corresponding to each cassette element. Another part underlined in the list is the sequence complementary to the gene-specific sequence.

**Table 3:** Yeast strains used in this study.

**Strains**	**Genotype**
**BY4741**	*MATa his3Δ1 leu2Δ0 met15Δ0 ura3Δ0*
**WXY3812**	BY4741 *UBC13-HA*
**WXY3814**	BY4741 *HA-UBC13*
**HK578-10A**	*MATa ade2-1 can1-100 his3-11*,*15 leu2-3*,*112 trp1-1 ura3-1*
**WXY3816**	10A *mCherry-UBC13*
**WXY3818**	10A *P*_*PGK1*_*-mCherry-UBC13*
**HK578-10D**	*MATα ade2-1 can1-100 his3-11*,*15 leu2-3*,*112 trp1-1 ura3-1*
**WXY3822**	10D *RAD5-sfGFP*
**WXY3824**	10D *P*_*PGK1*_*-RAD5-sfGFP*
**WXY3826**	10D *RAD5-mKikGR*
**WXY3828**	10D *P*_*PGK1*_*-RAD5-mKikGR*
**FYV1^*ɑ*^**	yRH182 *HHT1-sfGFP*

^***ɑ***^The detail information about strain yRH182 please see[2].

Next, we detected the expression of FP-tagged proteins by laser scanning confocal microscopy. The HHT1-sfGFP was controlled under its native promoter, and sfGFP fusion proteins were detected under the 488nm laser and located in the nucleus. All of the mCherry-Ubc13, Rad5-sfGFP, and Rad5-mKikGR fusion proteins only produced weak fluorescent signals. To enhance these signals, and further confirm the reuse of the *URA3* marker, we employed the plasmid pPUP, which contains a tandem *PGK1* promoter flanking the *URA3* gene [21], and used it to replace the native promoters. The procedure and results confirming the successful *PGK1* promoter replacement of the resulting strains are shown in S2 Fig.

**Fig 5:**
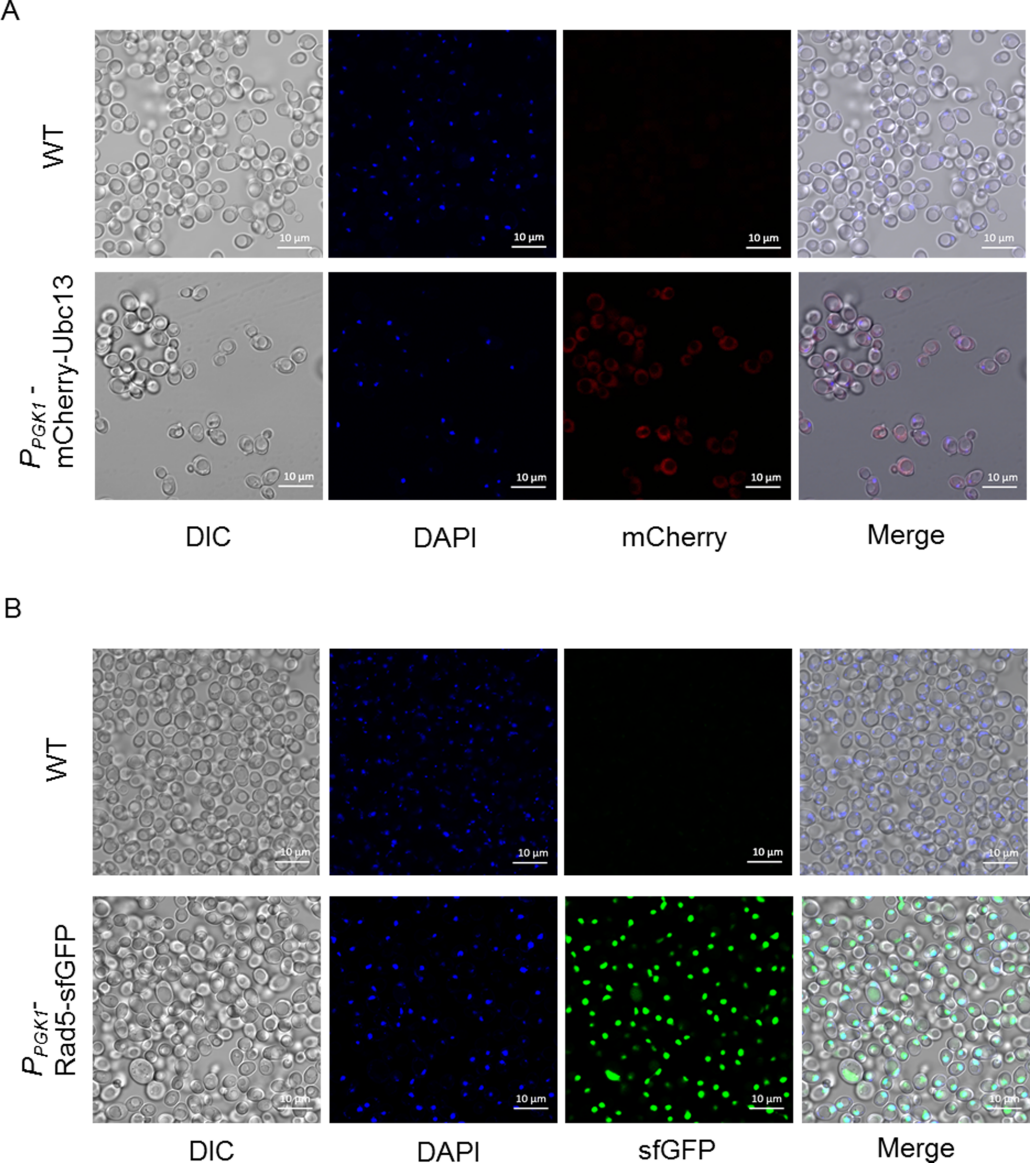
The subcellular localizations of mCherry-Ubc13 and Rad5-sfGFP. HK578-10A (upper A) and HK578-10D (upper B) are the wild-type yeast strains without modification; the tagged strains *P*_*PGK1*_-Ubc13-mCherry and *P*_*PGK1*_-Rad5-sfGFP are shown at the bottom of A and bottom of B, respectively. The nucleus was stained by DAPI. The images were obtained under Plan-Apochromat 63×/1.40 oil (Zeiss LSM780).

**Fig 6:**
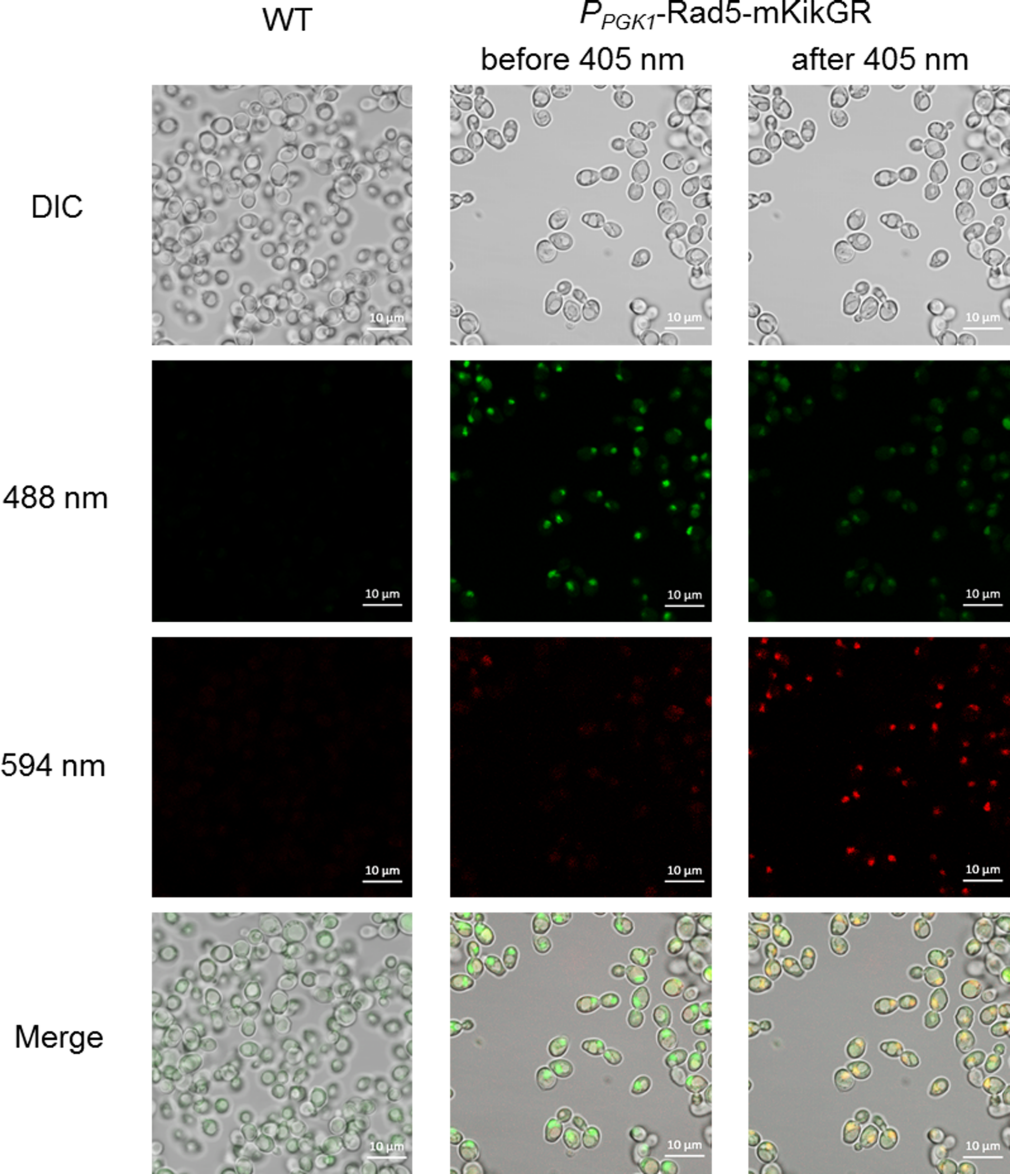
The photoswitching of mKikGR in *P*_*PGK1*_-Rad5-mKikGR strains. HK578-10D (left panel) is the wild-type strain without modification. The cells containing Rad5-mKikGR were imaged with 488-nm light and 594-nm light before (middle) and after (right) 405-nm laser photoswitching.

Under control of a constitutive *PGK1* promoter, we observed that the mCherry-tagged *UBC13* was located in both the cytoplasm and the nucleus (Fig 5A), which is accordance with controlling under its native promoter, as previously reported[5]. Similar to mCherry-Ubc13, the expression of the Rad5-sfGFP fusion protein was enhanced under the regulation of the *P*_*PGK1*_ promoter. Upon illumination with a 488-nm laser, the green fluorescence colocalized with the DAPI staining, indicating the nuclear localization of Rad5 (Fig 5B). This observation is consistent with the predicted function of *RAD5* in error-free DNA post-replication repair [29] and the early localization experiment[5]. The expression level of Rad5- mKikGR was also improved when driven by the *PGK1* promoter, since we detected a strong fluorescent signal in the resulting strains (Fig 6). To validate the photoswitching of Rad5-mKikGR, yeast cells were first observed under a 488-nm laser, which showed that the fluorescent green of Rad5-mKikGR was localized in the nucleus. After illumination with a pulse of 405-nm light, most of the signals of green fluorescence were converted into red fluorescence upon excitation with 594-nm light (Fig 6). Thus, our data demonstrate that all of these FP-labeled proteins of interest function normally in the resulting strains.

### Verification of epitope-tagging at the *UBC13* native genomic locus using biochemical analysis

**Fig 7:**
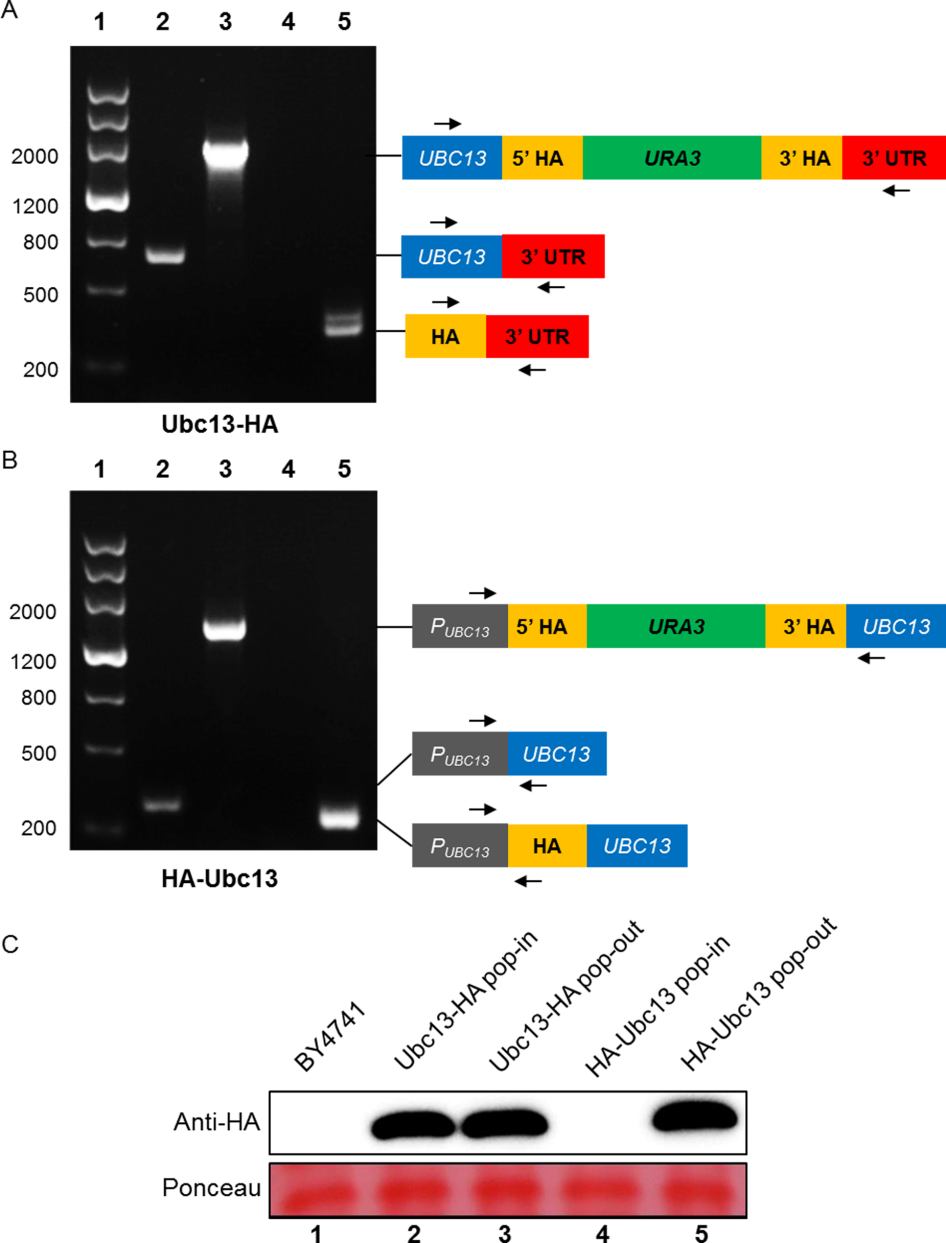
PCR and western blot analysis of HA-tagged Ubc13 yeast strains. (A) Ubc13-HA strain. (B) HA-Ubc13 strain. Lane 1: DNA ladder marker. Lanes 2 and 4: the original strain BY4741. Lane 3: the pop-in strain. Lane 5: the pop-out strain. Arrows indicate the locations of primers. The detail information of primers could be found in the part of yeast strains check primers in S1 Table. And the strains in each Lane 4 and 5 share the same pair of primers for analysis. (C) (Upper) Anti-HA antibody is used for detecting the HA tag. (Bottom) Ponceau S staining is shown as the total protein loading control.

Using the same technique as described above, the N- or C-terminals of *UBC13* tagged with HA stains were obtained separately (Table 3). As shown in Fig 7A and 7B, PCR analysis indicated that successful chromosomal integration had occurred as intended. The tagged proteins were examined by western blot analysis. The anticipated Ubc13-HA C-terminal fusion protein was detected in both pop-in and pop-out strains (Fig 7C, cf. lanes 2 and 3). In contrast, integration of the *HA-URA3-HA* cassette at the 5′ end of the *UBC13* ORF interfered with its expression (Fig 7B). Only if the cassette was popped-out were we able to detect the HA-Ubc13 N-terminal fusion protein by western blot analysis (Fig 7C, cf. lanes 4 and 5). Using a different strain, a 3×FLAG-Ubc13 N-terminal fusion cell line was created and the expression was verified by western blot (S3 Fig).

Compared with current *in vivo* protein-tagging techniques used in budding yeast, this strategy has three advantages. First, it allows tagging of N -terminals of nonessential genes at the native genomic locus, while the traditional methods only allow tagging of the C-terminal. Second, only the desired tag remains in the yeast genome after tagging, and the selection marker is recyclable. Third, the method described in this report does not alter the sequence of the 3′ noncoding region when C-terminal tagging is performed, meaning that alteration of the expression of the target gene is unlikely.

## Discussion

In this report, we describe a strategy to tag genes based on the *URA3* pop-in/pop-out system, which replaces “junk” tandem repeats with designed DNA elements for pop-out. As a proof-of-concept, plasmids containing three FPs and two epitope tags were constructed, each of which was demonstrated to tag Ubc13, Histone 3, or Rad5 at the N- or C-terminal successfully. We found that, consistent with the recent published study of tagging in yeast[30], 0.3~0.4 kb homologous sequences of the target intergration site is more efficient than 50 bp mentioned in the the previous epitope tagging studies[19, 31].

Yeast is a powerful eukaryotic model organism for biological and medical science. The development of many valuable tools in recent decades has made the genetic manipulation of budding yeast much easier. However, some limitations remain. For example, tagging of the N-terminal is one of the long-standing challenges in yeast genetics. In early studies, a tag could be introduced at the N-terminal of a gene of interest by promoter fusion; however, its native promoter had to be replaced because of the way in which the tag was integrated into the chromosome [32, 33]. The method we report here is suitable for N-terminal tagging of nonessential genes without any modification in the promoter region after integration. The limited number of selectable markers is another problem in yeast genetic engineering, especially when multiple genes are to be modified. Our *URA3* pop-in/pop-out system makes it possible to recycle a selectable marker; thus, one selectable marker can function repeatedly for multiple protein tagging, which is important for complex studies involving many proteins. For recycling of a selectable marker, pop-in/pop-out is the only strategy that has thus far been developed. However, in the pop-in/pop-out system, there is a problem that “junk” sequences such as *hisG* or *loxP* remain at the modification locus after popping out the selectable marker via HR. The efficiency of HR using limited homologous sequences, like PCR-mediated gene targeting, is clearly reduced when *hisG-URA3-hisG* is used more than once in the same strain because the *hisG* left in the genome causes significant internal homology [34]. Moreover, the unwanted “junk” sequence left in the chromosome might affect the expression of the target gene. For instance, a technique called DAmP (decreased abundance by mRNA perturbation) affects the expression of target gene through the disruption of natural 3′ untranslated region with an antibiotic-resistance marker insertion [35]. Thus, we here designed the DNA sequence from the tag itself for HR pop-out, which could avoid the decreased efficiency of HR and minimize the disturbance of the target gene’s expression level.

Labeling a protein of interest with an FP or epitope tag to study its properties, such as expression, localization, and dynamics, is widely used in biochemistry and cell biology. In addition, fluorescent probes are playing an increasingly important role in the newly developing techniques of single-molecule fluorescence detection and super-resolution microscopy [36, 37]. There are three main kinds of fluorescent probe: organic dyes, FPs, and quantum dots (QDs). Tagging of a protein with a fluorescent probe makes it possible to study the properties of the protein and its related biological processes at the single-molecule level [38, 39]. Using the photoconvertible protein mKikGR, a new powerful imaging technique, PhADE (photoactivation, diffusion, and excitation), was established and proposed to allow single-molecule study at physiological concentrations [37]. With the aid of anti-Flag-antibody-coated QDs, the mechanism by which CRISPR RNA-guided Cas9 interrogates DNA to recognize specific cleavage sites was clarified [40]. While the antibody for a protein of interest could be used for imaging at the single-molecule level [41], an epitope tag antibody would be more widely available from commercial sources. Taking these factors together, the convenient FP- and epitope-tagging techniques are helpful for solving scientific problems at the single-molecule level.

In summary, this protein-tagging method is an excellent tool for fusing an FP or epitope tag scarless to a target protein at its native locus in the yeast *S*. *cerevisiae*. Recently, Landgraf *et al* reported the same technique to tag the yeast genes with mNeonGreen and mCherry[30]. Here five tag constructs were presented and demonstrated to function in an easy-to-use and efficient way with homologous sequences. We believe that this strategy and the plasmids containing with newly developing fluorescent proteins will provide benefits in both bulk assays and single-molecule imaging in budding yeast research.

## Supporting information

S1 Fig The overlap PCR process. It details the steps for the *5’YFG-FP-URA3-FP-YFG3’*products. The *5’YFG(P*_*YFG*_*)* and *YFG3’(ORF*_*YFG*_*)* are PCR amplified from the yeast genome. The *FP-URA3-FP* cassettes are obtained by PCR from each plasmid. These three PCR products have a 25–30 bp duplication sequence (marked with wavy line) in the junction part for annealing and matching. With the mixture of these products as template, amplify to generate the final *5’YFG-FP-URA3-FP-YFG3’* products.
(PDF)

S2 Fig The PCR analysis of PGK1 promoter induced yeast strains. **(A)** *P*_*PGK1*_-mCherry-Ubc13 strain. The PCR products are 1.5 kb (lane 2), 3.9 kb (lane 3) and 2.2 kb (lane 4). The same pair of primers is used for PCR. **(B)** *P*_*PGK1*_-Rad5-sfGFP strain and **(C)** *P*_*PGK1*_-Rad5-mKikGR strain. The PCR products are 0.7 kb (lane 2), 3.0 kb (lane 3) and 1.4 kb (lane 4).
(PDF)

S3 Fig The PCR and western blot analysis of 3xFLAG-tagging Ubc13 yeast strains. **(A)** 3xFLAG-Ubc13 strain. Lane 1: DNA ladder marker. Lane 2: the original strains-HK578-10A. Lane 3: the pop-in strain. Lane 4: the pop-out strain. Arrows indicate the locations of primers. **(B)** (Upper) Anti-FLAG antibody is used for detecting the 3xFLAG tag (Bottom). Pgk1 was used as an internal control.
(PDF)

S1 Table Plasmid construction primers. (PDF)

S2 Table Promoter exchange primers. (PDF)

S3 Table Yeast strains check primers. (PDF)
