# Supplementary figures and images for: A method for labeling proteins with tags at the native genomic loci in budding yeast
Source: PLoS One. 2017 May 1;12(5):e0176184. doi: 10.1371/journal.pone.0176184 (PMC5411076; doi:10.1371/journal.pone.0176184)

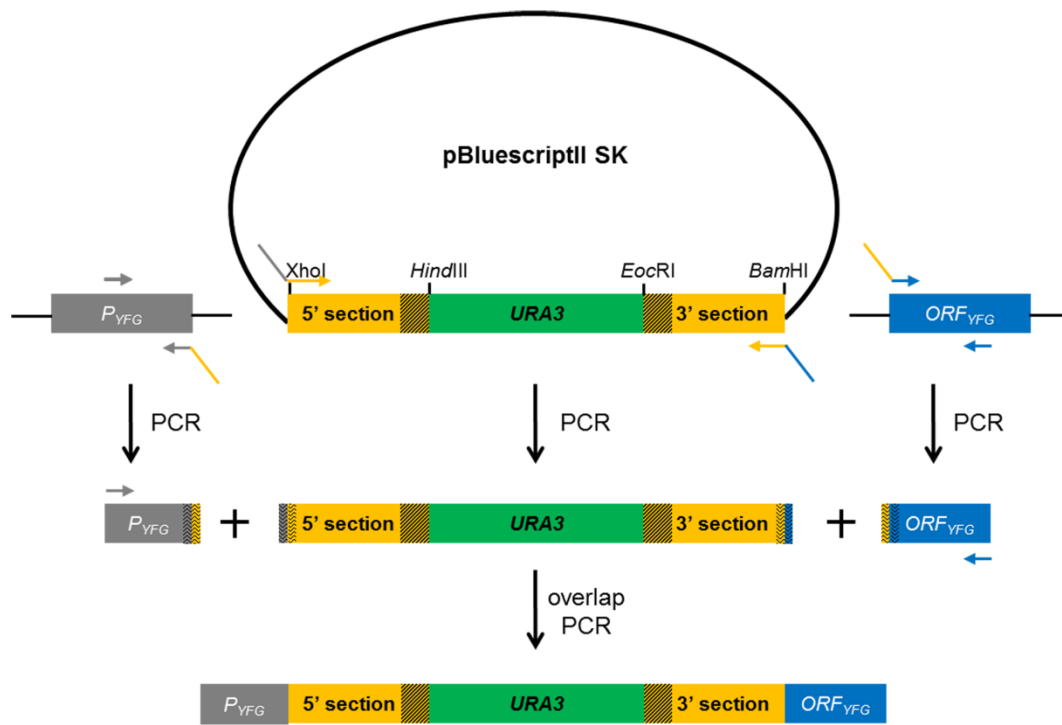

Supplement: S1 Fig — It details the steps for the 5’YFG-FP-URA3-FP-YFG3’products. The 5’YFG(PYFG) and YFG3’(ORFYFG) are PCR amplified from the yeast genome. The FP-URA3-FP cassettes are obtained by PCR from each plasmid. These three PCR products have a 25–30 bp duplication sequence (marked with wavy line) in the junction part for annealing and matching. With the mixture of these products as template, amplify to generate the final 5’YFG-FP-URA3-FP-YFG3’ products. (PDF) [file pone.0176184.s001.pdf]

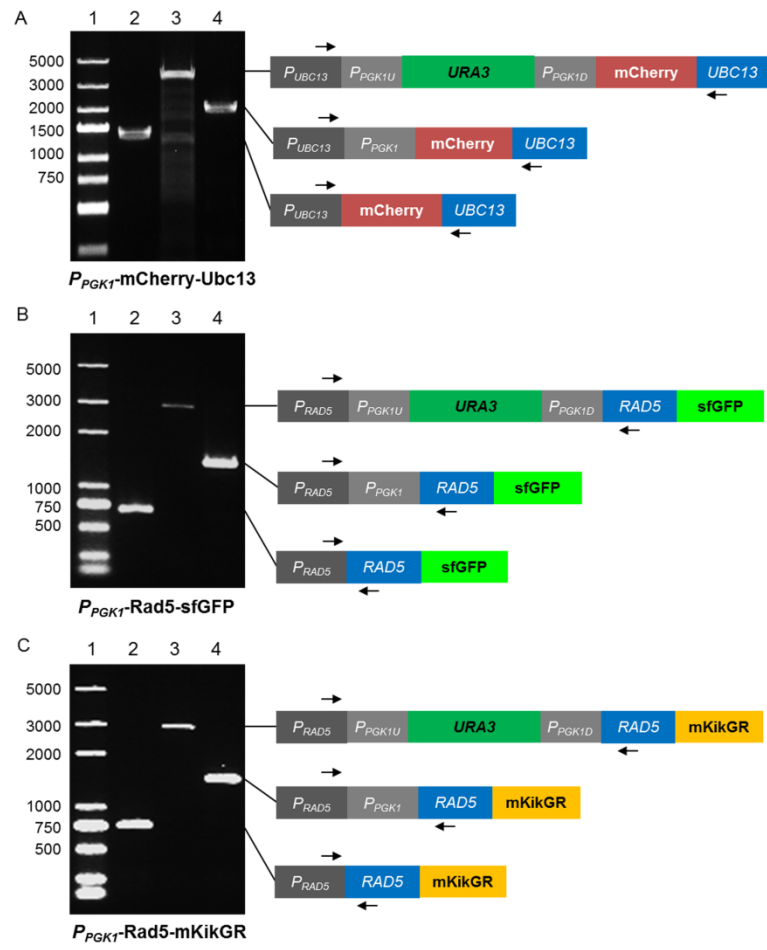

Supplement: S2 Fig — (A) PPGK1-mCherry-Ubc13 strain. The PCR products are 1.5 kb (lane 2), 3.9 kb (lane 3) and 2.2 kb (lane 4). The same pair of primers is used for PCR. (B) PPGK1-Rad5-sfGFP strain and (C) PPGK1-Rad5-mKikGR strain. The PCR products are 0.7 kb (lane 2), 3.0 kb (lane 3) and 1.4 kb (lane 4). (PDF) [file pone.0176184.s002.pdf]

A

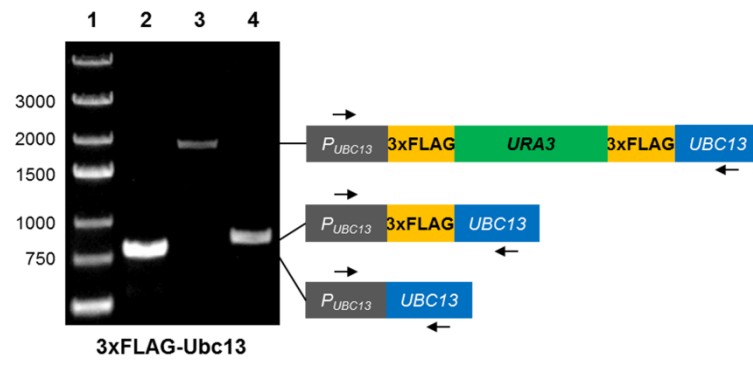

B

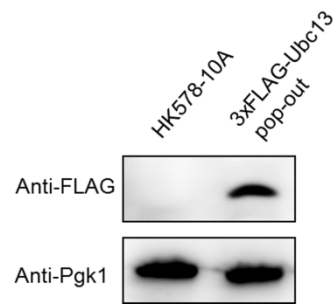

Supplement: S3 Fig — (A) 3xFLAG-Ubc13 strain. Lane 1: DNA ladder marker. Lane 2: the original strains-HK578-10A. Lane 3: the pop-in strain. Lane 4: the pop-out strain. Arrows indicate the locations of primers. (B) (Upper) Anti-FLAG antibody is used for detecting the 3xFLAG tag (Bottom). Pgk1 was used as an internal control. (PDF) [file pone.0176184.s003.pdf]
