# Supplementary material for: A method for labeling proteins with tags at the native genomic loci in budding yeast
Source: PLoS One. 2017 May 1;12(5):e0176184. doi: 10.1371/journal.pone.0176184 (PMC5411076; doi:10.1371/journal.pone.0176184)
Supplement: S1 Table — (PDF) [file pone.0176184.s004.pdf]

S1 Table. Plasmid construction primers

Plasmid construction Primers	Sequences (5’→3’)
**PHUH**	
pHA-URA3 F	TTCCCAAGCTTATGTACCCATACGATGTTCCAGATTACGCTGGGTAATAACTGATATAA
pHA-URA3 R	CCAAGAATTCAGCGTAATCTGGAACATCGTATGGGTACATAGCTTTTCAATTCAATTC
**pFUF**	
pFLAG-URA3 F	GATTATAAAGATCATGACATCGATTACAAGGATGACGATGACAAGGGGTAATAACT
pFLAG-URA3 R	ATGATCTTTATAATCACCGTCATGGTCTTTGTAGTCCATAGCTTTTCAATTCAATTC
pFLAG F	GAGCCACTCGAGATGGACTACAAAGACCATGACGGTGATTATAAAGATCATGAC
pFLAG R	GAGCCAGGATCCCTTGTCATCGTCATCCTTGTAATCGATGTCATGATCTTTATAATC
**pCUC**	
pURA3 F	CCAGTCAAGCTTGGGTAATAACTGATATAA
pURA3 R	GCCCAGGAATTCAGCTTTTCAATTCAATTC
pNmCherry F	GAACATCTCGAGATGGTGAGCAAGGGCGAG
pNmCherry R	CCAGTCAAGCTTCCTCTGCTTGATCTCGCC
pCmCherry F	GCCCAGGAATTCCAGTTCATGTACGGCTCC
pCmCherry R	GTGGATGGATCCCTTGTACAGCTCGTCCAT
**PGFUGF**	
pNsfGFP F	CGCCCTCGAGATGCGTAAAGGCGAAGAGC

pNsfGFP R	CCGCAAGCTTTTATTACGGTTACGAACTCCAGCA
pCsfGFP F	CGCCGAATTCGTGTTCAGTGCTTTGCTCGTT
pCsfGFP R	CCGCGGATCCTTATTATCATCATTTGTACAGTTC
**pKUK**	
pNmKikGR F	CGCCCTCGAGATGAGTGTGATTACATCAGAAATGAAGA
pNmKikGR R	CCGCAAGCTTTTATTATTATATTGTTTGTGGCGAGGCA
PCmKikGR F	CGCCGAATTCTTGTCGAATACCCAGAAGAAATAGT
PCmKikGR R	CCGCGGATCCTTATTAGGCTTCAAATTCATACTTGGCG
