# Supplementary material for: A method for labeling proteins with tags at the native genomic loci in budding yeast
Source: PLoS One. 2017 May 1;12(5):e0176184. doi: 10.1371/journal.pone.0176184 (PMC5411076; doi:10.1371/journal.pone.0176184)
Supplement: S2 Table — (PDF) [file pone.0176184.s005.pdf]

S2 Table. Promoter exchange primers

Promoter exchange primers	Sequences (5’→3’)
pUbc13-PUP F	TTAGCAAATAAGGTCAGGTTCATTGTAACATAGTTAGAATCGAGGTCGACGGTATCGAT
pPUP-mCherry R	GGCCATGTTATCCTCCTCGCCCTTGCTCACCATCTCGAGTGTTTTATATTTGTTGTAAA
pRad5up F	AAACCCCTCATTCTGGACCT
pRad5up-PUP R	CACTCTTTCCTTACTCACATTGTGATACTGTAAGACTT
pRad5up-PUP F	AAGTCTTACAGTATCACAATGTGAGTAAGGAAAGAGTG
pPUP-Rad5down R	TCCTGTTCAATATGACTCATTGTTTTATATTTGTTGTA
pPUP-Rad5down F	TACAACAAATATAAAACAATGAGTCATATTGAACAGGA
pRad5down R	TGATTCGGGGAAGACGGTAT
