# Supplementary material for: A method for labeling proteins with tags at the native genomic loci in budding yeast
Source: PLoS One. 2017 May 1;12(5):e0176184. doi: 10.1371/journal.pone.0176184 (PMC5411076; doi:10.1371/journal.pone.0176184)
Supplement: S3 Table — (PDF) [file pone.0176184.s006.pdf]

S3 Table. Yeast strains check primers

Yeast strains check primers	Sequences (5'→3')
**HA-Ubc13**	
pNCHK-UH F	GCCGCATCCGTATTGTTACCCGAA
pNCHK-UH R	ACGGCTCAGACGAAAACGTC
pNCHK-HA R	AGCGTAATCTGGAACATCGTATGGGTACAT
**Ubc13-HA**	
pCCHK-UH F	TGGCATTACAGCAGAACCACATGA
pCCHK-UH R	TTATGATCCGGAATTAAACG
pCCHK-HA F	ATGTACCCATACGATGTTCCAGATTACGCT
**3xFLAG/(pPGK1-) mCherry-Ubc13**	
pCHKUbc13 F	TTGAATGCAGAACAAGAAGG
pCHKUbc13 R	TCATATGGTGACTGTTCTGG
**Rad5-sfGFP/mKikGR**	
pCHKRad5 F	GGATATCCTGGAGAAAGAGC
pCHKRad5 R	GCATTCAAAATGGATTGAAC
**pPGK1-Rad5-sfGFP/mKikGR**	
pRad5up F	AAACCCCTCATTCTGGACCT
pRad5down R	TGATTCGGGGAAGACGGTAT
